# Evaluation of an Antimicrobial Stewardship Program for Wound and Burn Care in Three Hospitals in Nepal

**DOI:** 10.3390/antibiotics9120914

**Published:** 2020-12-16

**Authors:** Varidhi Nauriyal, Shankar Man Rai, Rajesh Dhoj Joshi, Buddhi Bahadur Thapa, Linda Kaljee, Tyler Prentiss, Gina Maki, Basudha Shrestha, Deepak C. Bajracharya, Kshitij Karki, Nilesh Joshi, Arjun Acharya, Laxman Banstola, Suresh Raj Poudel, Anip Joshi, Abhinav Dahal, Niranjan Palikhe, Sachin Khadka, Piyush Giri, Apar Lamichhane, Marcus Zervos

**Affiliations:** 1 Division of Infectious Disease, Henry Ford Health System, Detroit, MI 48202, USA; gmaki1@hfhs.org (G.M.); mzervos1@hfhs.org (M.Z.); 2 Kirtipur Hospital, Kathmandu 44600, Nepal; shankarrai1956@gmail.com (S.M.R.); dr.piyushgiri@gmail.com (P.G.); aparlamichhane@gmail.com (A.L.); 3 Kathmandu Model Hospital, Kathmandu 44600, Nepal; rdhojrajesh@gmail.com (R.D.J.); basudha111@gmail.com (B.S.); dahal.abhinav@hotmail.com (A.D.); niranjanpalikhe@gmail.com (N.P.); sachin_khadka18@hotmail.com (S.K.); 4 Pokhara Academy of Health Science, Pokhara 33700, Nepal; pahspokhara@gmail.com (B.B.T.); drarjunacharya@gmail.com (A.A.); lbanstola@hotmail.com (L.B.); poudelsuresh6@gmail.com (S.R.P.); anipjoshi@yahoo.com (A.J.); 5 Global Health Initiative, Henry Ford Health System, Detroit, MI 48202, USA; tprenti1@hfhs.org; 6 Group for Technical Assistance, Kathmandu 44600, Nepal; bajra.deepak@gmail.com (D.C.B.); k49karki@gmail.com (K.K.); jos_nil@live.com (N.J.)

**Keywords:** antibiotic resistance, stewardship, wound care, burn care, Nepal

## Abstract

Antimicrobial stewardship (AMS) programs can decrease non-optimal use of antibiotics in hospital settings. There are limited data on AMS programs in burn and chronic wound centers in low- and middle-income countries (LMIC). A post-prescription review and feedback (PPRF) program was implemented in three hospitals in Nepal with a focus on wound and burn care. A total of 241 baseline and 236 post-intervention patient chart data were collected from three hospitals. There was a significant decrease in utilizing days of therapy per 1000 patient days (DOT/1000 PD) of penicillin (*p* = 0.02), aminoglycoside (*p* < 0.001), and cephalosporin (*p* = 0.04). Increases in DOT/1000 PD at post-intervention were significant for metronidazole (*p* < 0.001), quinolone (*p* = 0.01), and other antibiotics (*p* < 0.001). Changes in use of antibiotics varied across hospitals, e.g., cephalosporin use decreased significantly at Kirtipur Hospital (*p* < 0.001) and Pokhara Academy of Health Sciences (*p* = 0.02), but not at Kathmandu Model Hospital (*p* = 0.59). An independent review conducted by infectious disease specialists at the Henry Ford Health System revealed significant changes in antibiotic prescribing practices both overall and by hospital. There was a decrease in mean number of intravenous antibiotic days between baseline (10.1 (SD 8.8)) and post-intervention (8.8 (SD 6.5)) (*t* = 3.56; *p* < 0.001), but no difference for oral antibiotics. Compared to baseline, over the 6-month post-intervention period, we found an increase in justified use of antibiotics (*p* < 0.001), de-escalation (*p* < 0.001), accurate documentation (*p* < 0.001), and adherence to the study antibiotic prescribing guidelines at 72 h (*p* < 0.001) and after diagnoses (*p* < 0.001). The evaluation data presented provide evidence that PPRF training and program implementation can contribute to hospital-based antibiotic stewardship for wound and burn care in Nepal.

## 1. Introduction

Antimicrobial resistance (AMR) has been recognized as a complex global health challenge lacking a universal solution. In 2015, the World Health Organization (WHO) released a global action plan (GAP) on AMR which provides a framework for developing national action plans on a country-by-country basis. The burden of health care associated infection is higher in low- and middle-income countries (LMIC) compared to higher income countries [1]. Inappropriate antimicrobial prescribing practices, lack of adequate antibiotic tracking systems, and limited healthcare funding to facilitate surveillance and laboratory infrastructure are some of the factors that have contributed to rising antimicrobial resistance in LMIC. There is evidence that antimicrobial stewardship (AMS) interventions are effective in increasing compliance with antibiotic policy, reducing duration of antibiotic treatment and potentially reducing hospital length of stay [2].

Post-prescription review and feedback (PPRF) programs include expert review of antibiotic prescribing decisions and feedback to the attending physician [3]. PPRF programs have been shown to be effective in U.S. hospitals, and on the basis of four studies reviewed by Dijck et al., there was a decrease in antibiotic days noted with audit and feedback in LMIC [4]. In addition, comparison of baseline and post-intervention data of a PPRF program in medical, obstetrics and gynecology, and general surgery wards at Kathmandu Model Hospital indicated decreased days of therapy per 1000 patient-days for courses of aminoglycoside and cephalosporin, increased justified use of antibiotics, de-escalation, and rational use of antibiotics [5].

To date, there are very limited data on the potential impact of AMS programs on antibiotic prescribing practices in burn and wound care centers in LMIC. Annually, an estimated 5 million deaths occur in LMIC due to injuries, with 10 to 50 times more individuals living with associated permanent disabilities [6]. Fire-related burns alone account for about 300,000 deaths annually, 95% percent of those occurring in LMIC. The highest rates of burns occur in Asia. In rural Nepal, burns are the second most common injury, accounting for 5% of disabilities. Overall, burns are the third most common injury after fall and road traffic accidents in the country [7]. Lack of a national burn registry makes estimating burden of disease difficult, however, a recent systematic review suggested the average hospital stay among burn victims ranged from 13 to 60 days in Nepal, with mortality estimates of 4.5 to 23.5% [8].

Studies have estimated infection-related mortality in burn victims to range from 40 to 60% [9,10,11]. Within LMIC, chronic wounds are commonly colonized and infected with antibiotic-resistant bacteria both during hospitalization and after discharge [12,13]. Inadequate hospital and clinic infection control protocols, delay in treatment, and use of self-treatment are some of the factors contributing to prevalence of infection in this population [14,15]. A primary concern is the increasing prevalence of antibiotic resistance in LMIC [16,17,18].

High morbidity and mortality associated with wounds and burns in Nepal and other LMIC has incentivized an urgent need to develop infection prevention practices and improve treatment. The aim of this study was to implement and evaluate the role of a hospital-based AMS program to support optimal antibiotic use for wounds and burns and in the longer-term decrease risks of infection from resistant pathogens.

## 2. Results

### 2.1. Patient Chart Data: Demographics

A total of 241 baseline and 236 post-intervention patient chart data were collected from the three study hospitals. At both baseline and post-intervention, a majority of patients were male and the mean age was less than 40 years. Number of study patients were evenly distributed across the three hospitals at both baseline and post-intervention. At post-intervention, there were more patient charts from the burn and less from the plastic and reconstructive surgery wards compared to baseline (*p* < 0.001). Length of stay decreased significantly within the burn unit (*p* < 0.001), as well as overall (*p* = 0.006) (see Table 1). There were no reported deaths among study patients.

### 2.2. Patient Chart Data: Antibiotic Use at Baseline and Post-Intervention

Overall, there was a decrease in mean number of intravenous (IV) antibiotic days between baseline (10.1 (SD 8.8)) and post-intervention (8.8 (SD 6.5)) (*t* = 3.56; *p* < 0.001). There was no significant change for mean number of oral (PO) antibiotic days between baseline (4.2 (SD 3.3)) and post-intervention (3.7 (SD 3.5)) (*t* = 0.66; *p* = 0.510).

Across the three sites, there was a significant decrease in mean days of therapy between baseline and post-intervention for both aminoglycoside (6.1 (SD 4.3) vs 4.6 (SD 2.1)) (*t* = 2.08, *p* = 0.04) and cephalosporin (6.0 (SD 6.1) vs. 4.1 (SD 3.4)) (*t* = 3.54, *p* < 0.001). There were no significant changes in mean days of therapy for quinolines, penicillin, metronidazole, and other prescribed antibiotics.

**Figure 1:**
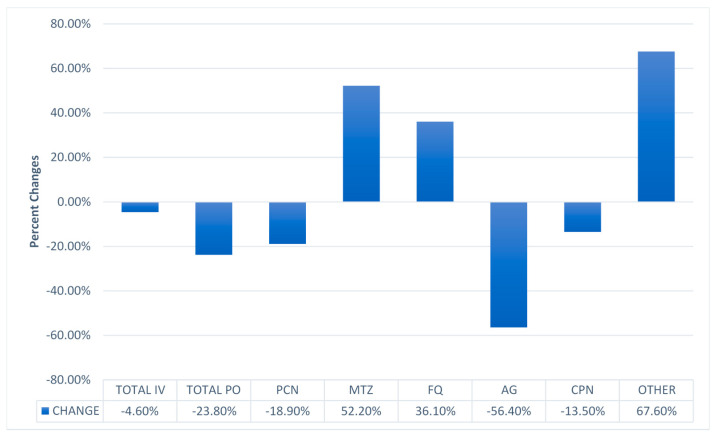
Changes in days of therapy (DOT) per 1000 patient-days (PD) between baseline and post-intervention in three hospitals in Nepal (Kirtipur, Kathmandu Model, and Pokhara hospitals). PCN = penicillin; MTZ = metronidazole; FQ: fluoroquinolone; AG: aminoglycosides; CPN: cephalosporin.

Utilizing days of therapy per 1000 patient days (DOT/1000 patient-days (PD)) for data across the three study sites, we found no change in administering antibiotics either IV (*p* = 0.67) or PO (*p* = 0.09). There was a significant decrease in use of penicillin (*p* = 0.02), aminoglycoside (*p* < 0.001), and cephalosporin (*p* = 0.04). Increases in DOT/1000 PD at post-intervention were significant for metronidazole (*p* < 0.001), quinolone (*p* = 0.01), and other antibiotics (*p* < 0.001) (Figure 1 and Table 2).

Looking at these data by site, we found no change in IV administration of antibiotics, but there was a decrease in administering PO antibiotics at Kirtipur Hospital (*p* = 0.004). Moreover, at Kirtipur Hospital, there was a significant decrease in use of cephalosporin (*p* < 0.001) but an increase in use of metronidazole (*p* = 0.002). At Kathmandu Model Hospital, there were significant decreases in use of penicillin (*p* = 0.04) and quinolones (*p* = 0.02), but a significant increase in use of metronidazole (*p* < 0.001). At Pokhara Academy of Health Science, there was a decrease in both cephalosporin (*p* = 0.02) and aminoglycoside (*p* < 0.001), but increases in quinolones (*p* < 0.001) and other antibiotics (*p* < 0.001). (Table 2).

An independent review conducted by infectious disease specialists at the Henry Ford Health System revealed significant changes in antibiotic prescribing practices both overall and by hospital. Over the 6-month post-intervention period, there was a noted increase in justified use of antibiotics, de-escalation, accurate documentation, and adherence to the study antibiotic prescribing guidelines at 72 h and after diagnoses (definitive) (Table 3).

Physician champions recorded information on recommendations made during the post-intervention period. Across the three study sites, there were a total of 249 logbook entries with 71 recommendations (28.5%). Among the recommendations, there were 53 cases (74.6%) in which the physician champion recommended a change in the antibiotic and 18 cases (25.4%) in which the recommendation was to stop antibiotics. Overall, 41/71 (57.7%) recommendations were followed by the prescribing physician. Among 47 entries with the reason listed for the recommendation, 25 cases (53.2%) were related to obtaining data on resistance/sensitivity patterns, 6 cases (12.8%) due to no definitive evidence of infection, and 5 cases (10.6%) were related to extended duration of antibiotic use. Other reasons included use of multiple antibiotics, IV to oral conversion, patient symptoms, and change from a broader to a narrower spectrum antibiotic.

## 3. Discussion

The World Health Organization Global Action Plan (GAP) for AMR includes five strategic objectives that must be addressed to decrease pathogen resistance to available pharmaceutical therapeutics. These objectives include to (1) increase awareness and understanding of AMR, (2) strengthen knowledge through surveillance and research, (3) reduce the incidence of infection, (4) optimize the use of antimicrobial medicines, and (5) ensure sustainable investment in countering antimicrobial resistance [18]. Over the past 5 years, the partnership between the Henry Ford Health System, Nepali private, public, and non-profit hospital systems, and the Group for Technical Assistance in Kathmandu has supported AMR stewardship education and programs. The data presented in this paper represent an important step for implementation of hospital-based stewardship programs with an emphasis on the urgent need in LMIC to reduce risks of infection in wound and burn care. The hospitals selected included the non-profit and government health sectors. Both Kathmandu Model and Kirtipur hospitals were part of a previous post-prescription review and feedback (PPRF) project, and all three hospitals remain connected to ongoing education and training through a new web-based program Global Learning in Antimicrobial Resistance (GLAMR).

The data presented provide evidence that PPRF training and program implementation can contribute to hospital-based stewardship in Nepal. Across all three hospitals, there is a clear indication that prescribing practices at post-intervention were more likely justified and followed antibiotic prescribing guidelines. Utilizing DOT/1000 PD analytics, we found that across the three hospitals there were significant decreases in the use of penicillin, cephalosporins, and aminoglycosides. Within each individual hospital, there was some variation in prescribing practices, however, there was evidence of decreased use of penicillin, aminoglycosides, quinolones, and cephalosporins. Variations may be attributable to differences in prescribing practices at baseline within the various study wards. A recent study published on bacteriological profile of burn wound infections in Nepal suggested predominance of resistant Gram-negative organisms such as *Acinetobacter* spp., *Pseudomonas* spp., and *Enterobacter* spp. [19]. The decrease in use of penicillin, cephalosporins, and quinolones is hence an encouraging signal towards recognition of bacterial epidemiology and appropriate use of antimicrobials. While there was an increase in metronidazole use at post-intervention, the overall increase in “justified use” of antibiotics in our post-intervention group indicates an overall improvement in prescribing practices. The use of metronidazole with another agent would have been deemed “unjustified”.

The changes across three hospitals within two separate locations in Nepal (Kathmandu and Pokhara) indicate that PPRF programs can be successfully implemented under different hospital administration and supports further dissemination as a part of hospital-based stewardship elsewhere in Nepal. Our evaluation of the previous implementation of the PPRF program at Kathmandu Model Hospital indicated decreases in use of cephalosporins and aminoglycosides. While there was no further decrease in these two antibiotics in the current study at Kathmandu Model Hospital, this may have been due to less use of these antibiotics at baseline.

The review of the prescribing practices shows significant improvements across all three study sites in terms of justified use, following guidelines, and documentation. Therefore, even as use of some antibiotics increased, these data suggest that prescribing practices at post-intervention were more likely appropriate to the diagnosis in terms of type and duration. Furthermore, the logbook data indicate that physician champions were actively reviewing patient charts within their wards and making recommendations. More than half of those recommendations were followed by the prescribing physician, with a majority of those recommendations due to information on pathogen resistance/sensitivity, lack of evidence of infection, and long duration of use of a single antibiotic.

The study strengths include the potential for introducing antimicrobial stewardship programs in low-resource hospital settings with comprehensive training and locally salient antibiotic prescribing guidelines. In addition, non-infectious disease physicians were successfully trained as physician champions and supported prescribing changes within their wards. Utilizing the existing training materials and guidelines reflective of potential regional differences in resistance and availability of antibiotics, the program can be duplicated elsewhere in Nepal. There are some limitations to the study. Data collection was dependent on manual review of handwritten notes given the lack of electronic medical record in Nepal. “Other courses” is an un-identified pool of antibiotics since the focus was on obtaining usage data on the most commonly prescribed antibiotics. Physician champions had to manually fill out log-books, which added to their workload, and hence not all data points were consistently entered. Out of a total of 249 log-book entries, only 47 outlined rationale for recommendations. The study length did not provide time to determine if there were any changes in resistance levels among pathogens.

## 4. Materials and Methods

The study was part of a larger AMR and AMS collaboration between the Henry Ford Health System Division of Infectious Diseases and Global Health Initiative (Detroit, MI, USA); the Group for Technical Assistance (Kathmandu, Nepal); and various non-profit, public, and private hospitals in Nepal. PPRF programs include expert review of antibiotic prescribing decisions and feedback to the prescribing physician. One strength of the current study is the focus of the PPRF program on wound and burn care. In many LMIC, there are inadequate numbers of infectious disease specialists, and therefore a key element of the adapted PPRF program in Nepal includes training “physician champions” in AMR and AMS. More details about the adaptation of the PPRF intervention for use in Nepal is described in detail elsewhere [5]. Variations for the current study included revisions to the antibiotic prescribing guidebook to include information on wound and burn care and a training-of-trainers approach, whereby the AMR “physician champions” were both trained in the PPRF program and provided with information and tools to train other healthcare providers within their wards. The antibiotic prescribing guidebook included 5 sections: (1) empiric guidelines, (2) suggested definitive guidelines with options depending on susceptabilities, (3) suggested duration of antibiotic therapy based on indications, (4) intravenous to oral conversions, and (5) renal dosing (Table 4). A total of 52 healthcare providers and hospital administrators including 6 physician champions from 3 study sites (Kathmandu Model, Kirtipur, and Pokhara hospitals) were trained over 2 days (11–12 July 2018).

### 4.1. Study Sites

**Figure 2:**
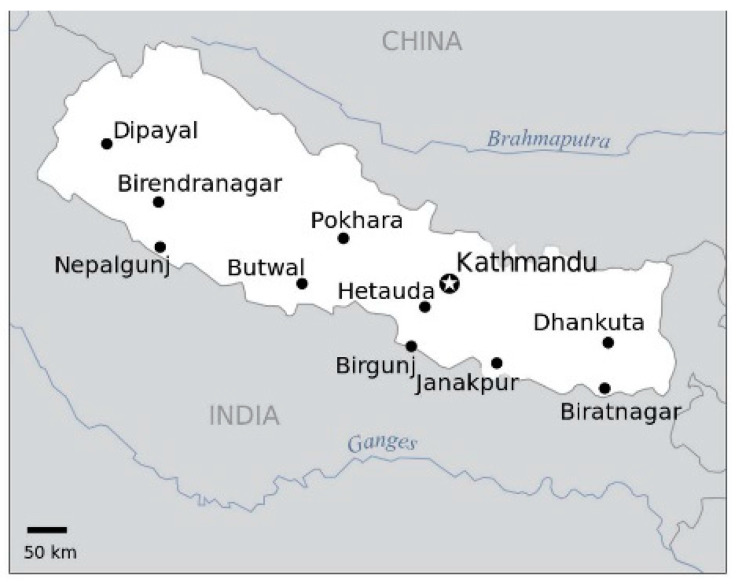
Map of major cities in Nepal.

The research took place in 2 hospitals in Kathmandu (Kathmandu Model Hospital and Kirtipur Hospital) and 1 hospital in Pokhara (Pokhara Academy of Health Science). Kathmandu is located in central Nepal and Pokhara is further toward the western region (Figure 2).

Both Kathmandu Model and Kirtipur hospitals are a part of a larger non-profit health organization, the Public Health Concern Trust (Phect, Nepal). Kathmandu Model is a 125-bed hospital that opened in 1993. Kathmandu Model Hospital provides a range of in-patient and out-patient services. The current study was focused in the general and specialized surgical wards. Kirtipur Hospital is a 100-bed hospital with additional specialized services including a 24 h emergency department. Kirtipur Hospital has a reconstructive surgery ward and the only burn intensive care unit in Nepal, which were the study sites for the current project. Kirtipur Hospital receives burn patients from throughout the country, and is part of the Resurge International Surgical Outreach Program that provides training and support to local hospitals and surgeons engaged in reconstructive and burn-related surgeries. Pokhara Academy of Health Science is a government facility and the second largest hospital in Nepal. The 500-bed hospital offers a broad range of services including a trauma center, burn unit, and surgical ward. These 3 wards were the study sites for the current project.

### 4.2. Study Population

The 3 study hospitals provide services to a range of socio-economic groups from both urban and rural areas in Nepal. Eligibility criteria for the patient chart review evaluation data included (1) inpatient within the study wards, (2) aged 15 + years, and (3) prescribed antibiotics for at least 72 h within the hospital.

### 4.3. Data Collection

#### 4.3.1. Patient Chart Data

Eligible patient chart data were collected for 6 months baseline (pre-PPRF training) from January 2018 to June 2018 and 6 months post-intervention between August 2018 and January 2019. The gap month (July 2018) was the implementation of the intervention. Data collection was coordinated and conducted by trained staff at a local nongovernmental agency (Group for Technical Assistance) located in Kathmandu.

Sample size was based on published data on duration of injectable drug use from Nepali hospitals [20]. Using a two-sided comparison of a continuous variable (days of therapy per 1000 study patient days, DOT/1000PD), we calculated that a sample size of 211 patients per group (baseline and post-intervention) was necessary to detect a difference of 20% between time periods (90% CI, α = 05).

Patient chart data included: (1) demographics (gender and age), (2) hospital/ward, (3) length of stay, (4) source of infection, (5) patient height/weight, (6) conditions present at study enrollment, (7) systemic antibiotic use during prior 72 h, (8) origin of onset of infection, (9) working and final diagnosis, (10) systemic antibiotic use throughout the hospital stay period, (11) therapy prescribed at discharge, (12) infection-related complications, (13) factors associated with persistent infection, and (14) disposition at end of hospital stay (if deceased, date and cause).

After data collection, patient chart data were reviewed by infectious disease specialists at the Henry Ford Health System to determine whether prescribed antibiotics were justified. Justification was determined by diagnosis, pathogen (when available), duration, and route (IV or PO) as described within the antibiotic prescribing guidelines (Table 4). Reviews included both initial therapy and therapy changes after recommendations by the physician champions.

#### 4.3.2. Physician Logbook Data

Physician champions were provided with antibiotic prescribing guidelines, which included a logbook. Through the logbooks, physicians documented chart reviews, recommendations made, and acceptance of recommendations by the prescribing physician. Recommendations were made verbally to the prescribing physician and/or as written notes. Logbooks were collected on a monthly basis to ensure that they were completed as required by the evaluation protocol.

### 4.4. Data Management and Analysis

Patient chart data were entered into REDCap (Research Electronic Data Capture) [21] by trained staff at the Group for Technical Assistance in Nepal. REDCap is a secure web application for building and managing online databases. REDCap allowed immediate access to the data both at the Henry Ford Health System and the project data team in Nepal. Data were reviewed and cleaned, which included deleting 2 cases that were under 15 years and 4 cases that were collected after the end of the 6-month post-intervention period. Continuous data were described using means and standard deviations, and univariate two group comparisons used independent two-group *t*-tests to assess significance. Categorical data were described using counts and percentages, and chi-squared tests were used to assess significance. Days of therapy DOT/1000 PD was calculated at baseline and intervention periods for IV and PO delivery and specific antibiotics. Days of therapy was calculated as 1921 at baseline (*N* = 241) and 1520 (*N* = 236) at post-intervention across the 3 sites. Days of therapy at baseline and post-intervention were calculated by site at 321 and 390 (Kathmandu Model), 358 and 365 (Kirtipur), and 592 and 449 (Pokhara), respectively. The proportion of DOT/1000 PD was compared between baseline and intervention time points using tests of proportion and Fisher’s exact test to determine significance. Statistical significance was set at *p* < 0.05.

Logbooks were collected by the project data team on a monthly basis and scanned. Scanned logbook data were sent to the evaluation team at the Henry Ford Health System for review. Scanned data were entered into Excel and analyzed using descriptive statistics. All statistical analysis was performed using SPSS 25.0 (Version 25.0. Armonk, NY, USA).

### 4.5. Ethical Review

The study was approved by the Institutional Review Board at the Henry Ford Health System, Detroit, MI (#11732), and the Nepal Health Research Council, Kathmandu, Nepal (#1523).

## 5. Conclusions

This study demonstrates successful implementation of PPRF as an antimicrobial stewardship tool at burn and wound in-patient centers in Nepal. There is an encouraging trend towards change in antimicrobial prescribing practice with more thoughtful and justified use.

## Figures and Tables

**Table 1:** Baseline and post-intervention patient characteristics, length of hospital stay, and distribution across hospital sites and wards.

Demographic Characteristics		Baseline	Post-Intervention	*p*-Value
Gender	Female	38.6% (93)	38.6% (91)	0.995
Mean age (SD)		39.2 (17.6) Range: 16–83	37.4 (17.4) Range: 15–88	0.252
Hospital	Kathmandu Model	33.2% (80)	31.5% (74)	0.837
	Kirtipur	32.4% (78)	34.9% (82)	
	Pokhara	34.4% (83)	33.6% (79)	
Ward	Surgery	67.4% (161)	65.5% (154)	<0.001
	Plastic and reconstructive surgery	22.6% (54)	8.9% (21)	
	Burn unit	10.0% (24)	25.5% (60)	
Mean length of hospital stay (days) (SD) by ward	Total	8.0 (5.9) Range: 3–48	6.4 (6.2) Range: 3–70	0.006
	Surgery	6.7 (3.7) Range: 3–27	6.6 (7.0) Range: 3–70	0.788
	Plastic and reconstructive surgery	8.3 (5.4) Range: 3–24	6.3 (6.0) Range: 3–27	0.181
	Burn unit	15.2 (11.2) Range: 3–48	6.1 (4.1) Range: 3–16	<0.001

**Table 2:** Days of therapy (DOT) per 1000 patient-days (PD) of prescribed antibiotics at baseline and post-intervention periods total and by study sites (Kirtipur, Kathmandu Model, and Pokhara hospitals).

Site	Antibiotic Delivery & Class	Baseline DOT/1000 PD (N)	Post-Intervention DOT/1000 PD (N)	*p*-Value
TOTAL SITES	Intravenous antibiotics	1165 (222)	1114 (227)	0.67
	Oral antibiotics	101 (46)	75 (31)	0.09
	Penicillin	301 (91)	241 (70)	0.02
	Cephalosporin	525 (167)	454 (167)	0.04
	Metronidazole	75 (30)	160 (56)	<0.001
	Quinolone	46 (17)	72 (24)	0.01
	Aminoglycoside	266 (84)	117 (39)	<0.001
	Other course	57 (16)	177 (53)	<0.001
KIRTIPUR	Intravenous antibiotics	292 (70)	304 (77)	0.56
	Oral antibiotics	64 (27)	37 (10)	0.004
	Penicillin	49 (15)	61 (19)	0.30
	Cephalosporin	264 (71)	228 (71)	<0.001
	Metronidazole	10 (4)	30 (10)	0.002
	Quinolone	9 (30)	14 (5)	0.40
	Aminoglycoside	8 (40	16 (4)	0.15
	Other course	18 (5)	16 (4)	0.73
KATHMANDU MODEL	Intravenous antibiotics	289 (70)	354 (72)	0.80
	Oral antibiotics	29 (17)	32 (18)	0.80
	Penicillin	63 (18)	54 (17)	0.04
	Cephalosporin	125 (63)	144 (61)	0.59
	Metronidazole	42 (18)	112 (42)	<0.001
	Quinolone	37 (14)	24 (9)	0.02
	Aminoglycoside	34 (21)	40 (16)	0.90
	Other course	20 (6)	16 (9)	0.23
POKHARA	Intravenous antibiotics	584 (82)	436 (77)	0.12
	Oral antibiotics	8 (2)	5 (3)	0.37
	Penicillin	189 (58)	127 (34)	0.22
	Cephalosporin	136 (33)	76 (35)	0.02
	Metronidazole	24 (8)	15 (4)	0.62
	Quinolone	0	33 (9)	<0.001
	Aminoglycoside	224 (59)	56 (18)	<0.001
	Other course	19 (5)	142 (41)	<0.001

**Table 3:** Justification, de-escalation, treatment rationale, and fidelity to guidelines at baseline and post-intervention by total sites and individual hospitals (Kirtipur, Kathmandu Model, and Pokhara).

Site	Review Criteria	Baseline	Post-Intervention	*p*-Value
TOTAL SITES	Was the antibiotics course justified? (Yes)	34.9% (84)	78.0% (184)	<0.001
	Were antibiotics de-escalated? (Yes)	28.0% (51)	85.9% (167)	<0.001
	Was the treatment rationale documented correctly? (Yes)	33.3% (62)	77.7% (146)	<0.001
	Were guidelines followed within the first 72 h of therapy? (Yes)	37.9% (67)	82.2% (143)	<0.001
	Were recommendations followed for definitive therapy? (Yes)	29.4% (50)	82.8% (154)	<0.001
KIRTIPUR	Was the antibiotics course justified? (Yes)	33.3% (26)	70.7% (58)	<0.001
	Were antibiotics de-escalated? (Yes)	41.8% (28)	87.5% (56)	<0.001
	Was the treatment rationale documented correctly? (Yes)	27.4% (20)	68.6% (48)	<0.001
	Were guidelines followed within the first 72 h of therapy? (Yes)	30.8% (20)	78.6% (44)	<0.001
	Were recommendations followed for definitive therapy? (Yes)	27.8% (20)	77.2% (44)	<0.001
KATHMANDU MODEL	Was the antibiotics course justified? (Yes)	46.3% (37)	77.0% (57)	<0.001
	Were antibiotics de-escalated? (Yes)	30.4% (21)	78.6% (55)	<0.001
	Was the treatment rationale documented correctly? (Yes)	53.5% (38)	81.4% (57)	<0.001
	Were guidelines followed within the first 72 h of therapy? (Yes)	63.0% (46)	80.6% (54)	0.016
	Were recommendations followed for definitive therapy? (Yes)	46.2% (30)	79.4% (54)	<0.001
POKHARA	Was the antibiotics course justified? (Yes)	25.3% (21)	86.1% (68)	<0.001
	Were antibiotics de-escalated? (Yes)	4.3% (2)	91.7% (55)	<0.001
	Was the treatment rationale documented correctly? (Yes)	9.5% (4)	85.4% (41)	<0.001
	Were guidelines followed within the first 72 h of therapy? (Yes)	2.6% (1)	88.2% (45)	<0.001
	Were recommendations followed for definitive therapy? (Yes)	0	91.7% (55)	<0.001

**Table 4:** Examples of empiric, definitive, and duration antibiotic prescribing guidelines.

**Empiric Guidelines**			
**Diagnosis**	**Suspected Pathogen**	**Empiric Therapy**	**Duration of Therapy**
Abdominal infection, community-acquired (e.g., cholecystitis, cholangitis, diverticulitis, abscess); NOTE: add gentamicin if MDRO suspected or identified	Enterobacteriaceae *Bacteroides* sp. Enterococci Streptococci	Preferred: • Ceftriaxone IV 1 g q24h + metronidazole IV or PO 500 mg q8h • +/− gentamicin IV 5 mg/kg q24h Alternative: • piperacillin/tazobactam IV 4.5 g q6h • cefepime IV 2 g q12h + metronidazole IV or PO 500 mg q8h + IV 5mg/kg q24hr • imipenem IV 1g q8h Oral options for outpatient therapy: • ofloxacin PO 400 mg q12h + metronidazole PO 500 mg q12h • moxifloxacin PO 400 mg q24h	4 days with adequate source control
**Suggested Definitive Guidelines**			
**Organism**	**Preferred Therapy**	**Alternative Therapy (Depending on Allergies and Susceptibilities)**	
*Enterobacter* spp. (AmpC-producing organism	Cefepime	Meropenem, colistin, tigecycline, trimethoprim/sulfamethoxazole, gentamicin, amikacin Consider combination therapy for extensively drug-resistant *Acinetobacter*	
**Suggested Duration of Antimicrobial Therapy Based on Indication**			
**Diagnosis**	**Duration of Therapy**	**Key References**	
Complicated intra-abdominal infection, community-acquired (appendicitis, cholecystitis, diverticulitis)	4 to 7 days after adequate source control	Infectious Diseases Society of America Guidelines: http://www.idsociety.org/uploadedFiles/IDSA/GuidelinesPatient_Care/PDF_Library/Intraabdominal%20Infectin.pdf Other resources: http://www.nejm.org/doi/pdf/10.1056/NEJMoa1411162	
